# The Training of the Hands

**Published:** 1895-04-06

**Authors:** 


### Modern Sociology.

### THE TRAINING OF THE HANDS.

More and more does technical education force its way to the front. For a time it was thought that intellectual training was everything, as indeed it might be if all men were writers, or physicians, or lawyers, but for those who till the soil, or dig out coals from beneath its surface, or weave, or forge, or build, there is but little guidance to be had from a knowledge of the geography of Patagonia, or the date of the Punic war. A blunder was made, though now largely corrected, in transforming the workman into the student, instead of trying to fuse the two into one. The workman who knows the principles of his craft, who works by science instead of rule of thumb, will succeed better than the one who has had no such training, and will probably make a better living than the man who can work only with books and ink. Great is the multiplication table, but it does not contain the whole sum of human knowledge, the full satisfaction of human need. Not that we would despise the mental development given by purely intellectual studies, but it is an error to think that it is the whole of education for every man.

Technical education, born in distant Finland, is conquering the civilised world, but varying its modes in every country. In Britain it means, speaking generally, the habit of using tools and gaining general handiness rather than any special preparation for a given trade. Even when it helps a journeyman to acquire the higher branches of his trade, it does not, as a rule, exactly fulfil the rôle of the "apprenticeship schools" of the Continent. There a boy goes to one of these institutions with a view to learning his chosen trade on both its scientific and practical sides, or perhaps to going through various kinds of workshops to find out what line he should finally adopt. Thus at the Diderot School in Paris the pupils during the first year go through a training in both wood and iron work. After this they can select any section of either industry for a special subject of study. The general "handiness" is gained in the first or experimental year, and it would certainly be a blunder to regard that year as wasted, because it is not spent in apprenticeship to a given calling. Yet unless special training for special work is in view it is to be feared that poor parents will not see the use of making any sacrifices for the sake of giving their children this instruction, and technical education will sink down into a luxury for the amateur. The first need of every man is the power to earn a living, and education which ignores this will fail in its object.

It is probable that this distinctly practical side of technical education is better kept in view abroad than in England. Thus at the Horological School at Waltham, U.S., the pupils are taught not only how to make the Waltham watches, but are instructed in the mechanism of those of Swiss and English make, so that they can repair any watch entrusted to them, whencesoever it came originally. A parallel case of the thorough instruction of girls is found in the Industrial School at Verviers, in Belgium. The special industry of the neighbourhood is weaving, and young girls who are employed in the weaving mills are taught darning on their only free day, Sunday. This is not the rough and ready darning of the home circle, but a kind necessary in their work. "The lessons are admirably thorough and practical," says an American observer, "the mended places in fabrics being indistinguishable from new cloth, while pieces cut out completely are so skilfully restored as to deceive closest observers." The school, however, does not confine itself to such merely mechanical training. Physics, mechanics, and chemistry are also taught, but with direct application to local industries. Chemistry is especially brought to bear on dyeing, and drawing on the composition of designs for various fabrics. The instruction, in short, is meant to train workmen who not only can carry out the processes of their special industry, but know why they do so.

Processes are mechanical and limited in operation. A knowledge of the principles underlying them gives the power of endless variation. Take an example from a Parisian dressmaking school. "A young girl, selected from a section of eight or ten, was shown a fashion plate of costumes, and told to sketch one on a blackboard from memory, at the same time adapting it to a very stout girl, who was chosen as the person for whom the costume was to be made. This she did, referring only twice to the fashion plate for an instant. Then the measure was taken, and the cutting and basting done by the rest of the section. In twenty minutes' time a costume was completed, which was an exact reproduction of that sketched upon the blackboard."

But why, some old-fashioned people ask, can this not be taught in an ordinary workshop? The illogical but practical answer is, "Because it isn't." We have no longer the master handicraftsman, whose one or two apprentices share with him every part of his work, but the manufacturer, each of whose paid hands can perform some small section perfectly, but could in no case go through the whole process. The unintelligent workman is content with this fractional perfection; the intelligent one is not, for he knows that without a wider knowledge of the whole work no advancement of any kind is possible. Manufacturers also are beginning to find out that the man who understands a little more than just what he has to do is the most valuable worker. In Belgium, at least, they are often the supporters of the technical schools. In France the municipality contributes, and in both countries the fees are very small. Where the course of technical instruction is long—and in some schools the curriculum demands three or four years' attendance—concurrent instruction of a more purely abstract kind is given, so that general intelligence is not sacrificed to special development.

But still there are many parents who cannot afford to send their children to a technical school; even the smallest fees are too high for them. This objection cannot be entirely got over, and Mr. Pratt, the founder and chief supporter of the Pratt Institute at Brooklyn, declares that while he would willingly admit pupils gratuitously, he has found that instruction is more appreciated when some payment is made for it. On the other hand, the municipality of Paris thinks it worth while not only to give gratuitous instruction, but to give a small payment to students in some of its schools. Perhaps Paris is not far wrong in so thinking, for the worth of a trained workman to his town and nation is far more than the amount it takes to educate him.

